# Viral-Induced Mortality of Prokaryotes in a Tropical Monsoonal Estuary

**DOI:** 10.3389/fmicb.2017.00895

**Published:** 2017-05-23

**Authors:** Vijayan Jasna, Ammini Parvathi, Angia Sriram Pradeep Ram, Kizhekkapat K. Balachandran, Nikathil V. Madhu, Maheswari Nair, Retnamma Jyothibabu, K. Veeraraghava Jayalakshmy, Chenicherry Revichandran, Télesphore Sime-Ngando

**Affiliations:** ^1^CSIR-National Institute of Oceanography, Regional Centre (CSIR)Kochi, India; ^2^Laboratoire Microorganismes: Génome et Environnement, UMR Centre National de la Recherche Scientifique 6023, Université Clermont-AuvergneAubière, France

**Keywords:** diel variations, viruses, prokaryotes, viral lysis, bacterial carbon demand, bacterial-viral interaction, Cochin estuary

## Abstract

Viruses are recognized as the most abundant and dynamic biological entities in the marine and estuarine environment. However, studies on the dynamics and activity of viruses in transient estuarine systems are limited. This study examines temporal and spatial variations in viral abundance (VA) and viral activity across the salinity gradient in a monsoon-driven tropical estuarine system (Cochin estuary, CE) along the southwest coast of India. Water samples were collected from five stations (with different hydrological settings) every 3 h for 24 h period during two distinct seasons, namely pre-monsoon (PRM, dry season) and monsoon (MON, wet season). Time series measurements were made for a spring and neap tidal cycle for each season at all the stations. The results showed marked spatial and seasonal variability with relatively low diel and tidal variations in VA and lytic activity. Viral activity was found to be distinct in five stations studied with the maximum activity in the mesohaline regions (salinity <20) of the estuary. This region was characterized by high VA, lytic infection and viral production, accompanied by low (BGE) and high bacterial respiration. Based on viral lytic production, lytic viruses were found to be responsible for the release of ca. 72.9 ± 58.5 μg C L^−1^d^−1^ of bacterial carbon. The contribution of the viral shunt to the dissolved organic carbon (DOC) pool was higher during the dry season (PRM) than MON. Statistical analysis confirmed a significant association of viruses with the host availability and salinity. This work demonstrates the spatiotemporal distribution of viruses in a tropical estuarine ecosystem and highlights their role in microbial mortality across different salinity gradients. This study forms the first report on viral processes from a monsoon-driven tropical estuarine ecosystem.

## Introduction

Viruses are the most abundant and diverse biological entities that form an integral component of the microbial food web in marine, estuarine, and freshwater ecosystems (Weinbauer, 2004; Suttle, 2007; Sime-Ngando, 2014). They are important controlling agents of the microbial community composition, diversity and succession (Weinbauer and Rassoulzadegan, 2004) and have been shown to play key roles in nutrient cycles (Fuhrman, 1999; Weitz and Wilhelm, 2012). As obligate parasites, viruses are dependent upon their hosts for their growth, replication and assembly. Hence, the physiological state and metabolism of host cells can greatly influence viral abundance and production. New viral progeny released to the environment is presumably exposed to various environmental factors, which may reduce infectivity, degrade or remove virus particles and adversely affect adsorption onto the host, thereby reducing the chances of a successful host encounter and infection (Mojica and Brussaard, 2014). Most importantly, the niche of viruses can be constrained either by their hosts or by their own sensitivity to variations in ionic strength and temperature. Therefore, both abiotic (like temperature, salinity, phostosynthetic active radiation, UV, pH, inorganic and organic particles, nutrients) and biotic factors (like prokaryotes, grazers, chlorophyll *a*) govern viral dynamics in aquatic systems (Mojica and Brussaard, 2014).

Viral abundance can fluctuate over shorter time scales such as hourly or daily in response to changes in viral production (Winget and Wommack, 2009; Winget et al., 2011; Ni and Zeng, 2016). Viral processes are host dependent and hence its temporal variability is linked to activity and productivity of bacterioplankton (Jiang and Paul, 1994; Winter et al., 2004) and specific bacterial groups (Dolan and Simek, 1999; Bettarel et al., 2011). Short term variations in bacterioplankton are well-documented. Many studies that has focussed on understanding the short term variations in bacterioplankton production showed different peaks in production ranging from the early evening (Jugnia et al., 2000), or late night, or early morning (Canon et al., 1998), or during midday (Gasol et al., 1998), or varies little (Torreton, 1999) or irregularly (Sherr et al., 2001). Diel variations in bacterioplankton is by and large attributed to light which may influence the bacterial production through photosynthetic production of dissolved organic carbon (DOC), through DNA damage (Booth et al., 2001) or indirectly through viral inactivation, destruction or protistan grazing (Wommack et al., 1996; Mojica and Brussaard, 2014). Seasonal patterns in viral abundance demonstrated highest abundance in summer and lowest in winter (Jiang and Paul, 1994). Several controlling mechanisms of viral processes such as trophic/nutrient level (Danovaro et al., 2003) and salinity (Jiang and Paul, 1994) has been reported. However, conflicting conclusions about controlling factors are drawn by different researchers in marine environments suggesting that these mechanisms are complex and multi-factorial.

During the last two decades, much attention has been given to viral processes in marine coastal and oceanic waters and, to a lesser extent, to transition zones, such as tropical estuaries. Estuaries are one of the most biologically active environments of the biosphere, characterized by varying temperature and nutrient concentrations and steep salinity gradients. Estuarine environments have been of great interest to microbial ecologists due to their high productivity and exploited aquatic habitats (Wommack et al., 1992; Winget and Wommack, 2009; Winget et al., 2011; Ni et al., 2015). The mixing front between marine tidal water and riverine discharge is an area of dramatic changes which can trigger important physiological, genetic, and ecological shifts in microbial hosts (del Giorgio and Bouvier, 2002). The virioplankton distribution in estuarine systems (Charente estuary, France) is reported to be entirely driven by mixing processes and hydrodynamic conditions (Auguet et al., 2005). In estuarine systems, seasonal variations in nutrient availability stimulates switch over between classical microbial loop (nutrient enrichment) and viral loop (nutrient depletion) (Montanié et al., 2014). However, little is known on the viral-mediated processes in these regions, particularly from the virio-ecological perspective in tropical estuarine systems impacted by monsoon cycles.

Cochin estuary (CE), is one of the most productive estuary along the southwest coast of India characterized by freshwater input during monsoonal rains (Jyothibabu et al., 2006). CE is connected to the Arabian Sea through two inlets, namely Cochin (width 450 m) and Azhikode (250 m) with channel depths of 5 and 14 m respectively. Heavy rains keep the estuary freshwater-dominated for 6 months during monsoon (wet season) and seawater-dominated for the rest of the period (dry season). The southwest monsoon has considerable influence on the physicochemical and biological characteristics of the estuary (Jyothibabu et al., 2006). The estuary receives riverine freshwater discharge (2.0 × 10^10^ m^3^yr^−1^) from six rivers namely, Periyar, Pampa, Achankovil, Manimala, Meenachil, and Muvattupuzha (Srinivas et al., 2003). Riverine discharges along with tidal propagation and prevailing current patterns makes this estuary a very complex system. The tides (amplitude ~1 m) in the CE are mixed semi-diurnal, which get modified inside the estuary to create different hydrologic zones. Unlike other estuaries in the west coast of India, the tides in Cochin estuary are forced from two inlets and the estuarine morphology is complex with many inter-connected channels and rivers (Jyothibabu et al., 2006). The entire estuary is dominated by seawater during pre-monsoon except for the upstream (toward south) Vembanad Lake region. The variations in salinity due to tides, riverine runoff or due to the closure of an artificial barrage situated upstream have considerable influence on the distribution of chemical and biological parameters within the estuary (Jyothibabu et al., 2006).

Recent studies in CE have indicated that increased microbial activity has transformed the estuary from an autotrophic to heterotrophic system (Gupta et al., 2009). Parvathi et al. (2015) have reported bacteria to be the predominant hosts for viral proliferation in this estuary. However, the influence of salinity fluctuations due to tidal and monsoonal cycles on viral lysis and bacterial dynamics in CE is largely unknown. The present study was conducted with the objectives to understand the diel, tidal, spatial and seasonal (dry and wet) variations in viral processes in CE, along with physicochemical variables to better understand the controlling mechanisms of viral dynamics.

## Materials and methods

### Study sites and sampling

**Figure 1:**
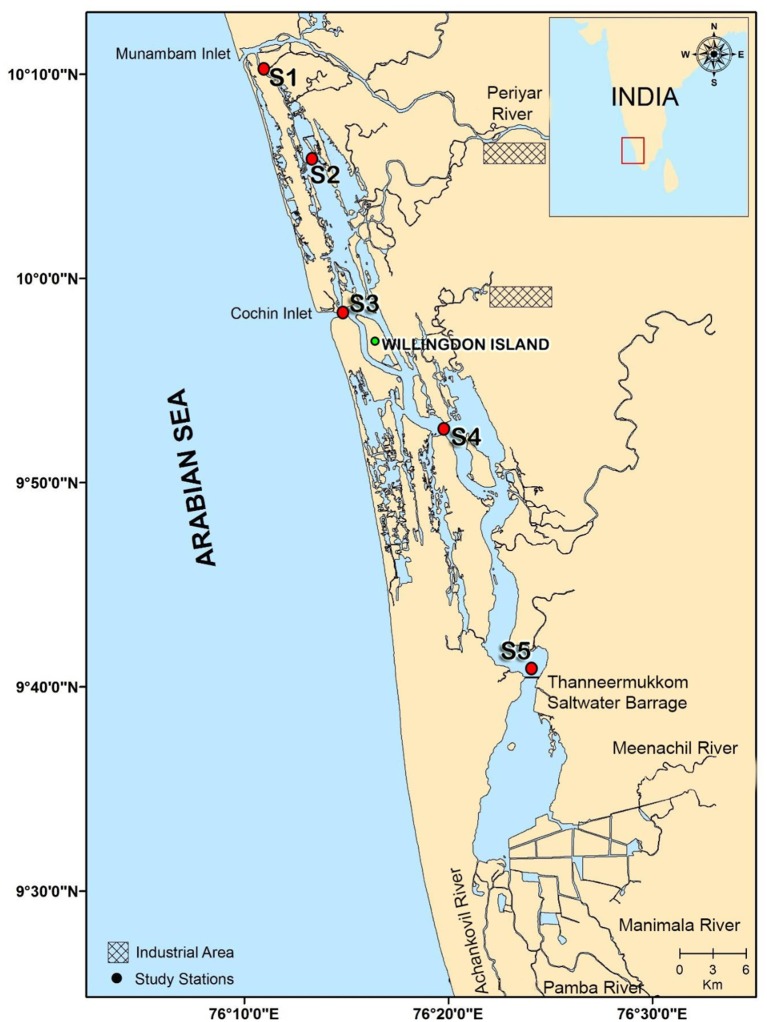
**Station locations in the Cochin estuary (CE)**. Stations 1 and 3 are the two inlets. Station 2 lies in between the two inlets toward the north of the estuary, stations 4 and 5 lies toward upstream (south) of the estuary.

Five stations in the Cochin estuary (CE; Figure 1) were selected based on the salinity regimes to perform 1 diel cycle measurement. The measurements were made at every 3 h interval for 24 h during spring and neap tide phases in two distinct seasons, monsoon (MON; July–August) and pre-monsoon (PRM; March–April) in 2009. Stations 1 and 3 (S1 & S3) represent the two inlets, Station 2 (S2) is located midway between the inlets, and Stations 4 and 5 (S4 & S5) are located toward the southern side /upstream of the Cochin inlet (S3).

Water samples from the chosen stations were collected using a 5L Niskin sampler (Hydro-Bios, Germany) from the surface and bottom of the water column during MON (July–August) and dry PRM (March–April) seasons. Water depths at all the stations were 1–2 m, except for S3, where the depth was 12 m. Samples were taken at mid depth from S3 alone. Water samples were transferred to sterile containers and transported to the laboratory in refrigerated boxes within 1 h of collection and processed immediately upon arrival to laboratory.

### Physico-chemical parameters

Temperature and salinity were measured using a conductivity temperature density profiler (CTD, SBE Seabird 19; accuracy ± 0.001°C for temperature and ± 0.001 S/m for conductivity). Dissolved oxygen (DO) and Biological Oxygen Demand (BOD) were estimated according to Winkler's titration method (Grasshoff, 1999). For the BOD estimation, the water sample was collected in a 300 ml stoppered glass bottle without trapping any air bubble, wrapped with black paper and incubated for 5 days at 20°C. The dissolved oxygen remaining in the bottle was then fixed with Winkler reagents and analyzed in the same manner as that of DO. BOD of the sample was calculated according to Grasshoff, 1999). Dissolved inorganic nutrients such as ammonia (NH_4_-N), nitrite (NO_2_-N), nitrate (NO_3_-N), phosphate (PO_4_-P), and silicate (SiO_4_-Si) were analyzed spectrophotometrically according to standard procedures (Grasshoff, 1999). Chlorophyll *a* (Chl *a*) concentrations were determined fluorometrically from 500 ml of water samples collected on GF/F filters (0.7 μm, Whatman, USA). The pigments were extracted in 90% acetone overnight in the dark at 4°C. The supernatant was used to determine the functional Chl *a* pigments (Parsons et al., 1984).

### Abundances of viruses and prokaryotes

For enumeration of viruses (VA) and prokaryotes (PA), water samples (in triplicates) were fixed immediately with 0.02 μm filtered, buffered formalin (2% *v/v*). Subsamples of 1–2 ml were filtered (<15 KPa vacuum) through 0.02 μm pore-size Anodisc filters (Whatman, USA) and stained with 1:400 diluted SYBR green I (Invitrogen, CA, USA) as previously described (Patel et al., 2007). The filters were air dried on absorbent paper and mounted between a slide and a glass cover slip with a special antifading mountant [50% glycerol, 50% PBS—phosphate buffered saline (0.05M Na_2_HPO_4_, 0.85% NaCl, pH 7.5), 0.1% p-phenylene diamine] and the virus-like particles were enumerated under epifluorescence microscope (Olympus BX 41, Olympus, USA). Prokaryotes were distinguished from virus-like particles (VLPs) on the basis of their relative size and brightness (Patel et al., 2007). A blank was routinely examined as a check for contamination of the equipment and reagents.

The total viable counts (TVC) were used to estimate the number of physiologically active bacteria (Joux and Lebaron, 1997). Briefly, 5 ml of the water sample (in triplicates) was mixed with 50 μl of 0.05% yeast extract, 50 μl of antibiotic cocktail (nalidixic acid, pipemidic acid, piromidic acid, and cephalexin) and incubated in the dark for 6 h. The sample was fixed in 2% formalin (final concentration) and bacteria were enumerated under epifluorescence microscope after staining with acridine orange (0.1 g/100 ml). Swollen or elongated cells were only considered as viable bacteria for enumeration.

### Heterotrophic bacterial production, respiration, and growth efficiency

Heterotrophic Bacterial Production (BP) was determined by incorporation of labeled thymidine (^3^H-TdR) into bacterial DNA (Fuhrman and Azam, 1982). Briefly, a 30-ml water sample (in triplicates) along with trichloroacetic acid (TCA) killed control (1% final concentration) were inoculated with labeled thymidine (specific activity 18 Ci/mmol, BARC, Mumbai, India) at a final concentration of 10 nM. Previous experiments from CE with varying concentrations from 1-100 nM revealed maximum incorporation at 10 nM concentration (data not shown). Samples were incubated in the dark for 60 min at *in situ* temperature. TdR incubation was stopped by adding 1% TCA (final concentration). The samples were filtered through 0.22-μm membrane filter, extracted twice with 5% ice-cold TCA, and rinsed with 80% ethanol. The dried filters were placed in scintillation vials and 0.5 ml of ethyl acetate was added to solubilize the filters. This was followed by addition of 5 ml dioxin-based scintillation cocktail (SRL Chemicals, Mumbai, India). Radioactivity was measured using a liquid scintillation counter (Beckman Coulter, LS 6500, USA). BP, calculated in moles of TdR incorporated into DNA, was converted into the number of bacterial cells produced by applying a conversion factor of 1.96 × 10^18^ cells mol^−1^ (Pradeep Ram et al., 2007) and to carbon using a factor of 2 × 10^−14^ gC cell^−1^ (Bell, 1993).

For bacterial respiration (BR), water samples (in duplicates) were filtered through 0.8 μm filter to remove large phytoplankton and bacterial grazers. Filtration was carried out under low differential pressure (<50 mm Hg) to avoid disruption of fragile cells. Water samples were collected in six 300 mL BOD bottles. Time zero control samples (in triplicate) were immediately fixed with Winkler's reagents. Another set of samples (in triplicate) were maintained at *in situ* temperature in the dark for 24 h before fixation. The difference in consumption of dissolved oxygen in the bottles was used to estimate the respiration rates.

Bacterial growth efficiency (BGE) was derived as the slope of bacterial production vs. sum of bacterial production and bacterial respiration [BGE = BP/(BP + BR)] and expressed as a percentage (Pradeep Ram et al., 2007). The bacterial carbon demand (BCD) was calculated as the sum of BP and BR (del Giorgio et al., 2011).

### Viral production (VP) and turnover rates

To estimate VP, the dilution technique described by Wilhelm et al. (2002) was used. Briefly, 100 ml water sample was diluted with 3 volumes of virus-free (0.02 μm pore-sized pre-filtered) water and incubated in the dark. Subsamples (1 ml; in triplicates) for bacterial and viral abundances were taken at every 3 hourly intervals up to 24 h and the counts were determined by epifluorescence microscope (see previous section). VP rates were determined from the first order regression of viral abundance versus time after correcting for the loss of the bacterial hosts between experimental samples and natural samples. VP was calculated as VP = m × (B/b) (Hewson and Fuhrman, 2007), where “m” is the slope of the regression line, “b” is the concentration of bacteria after dilution and “B” is the concentration of bacteria prior to dilution. Viral turnover rates were estimated by dividing viral abundance by VP rates.

### Viral-mediated mortality of bacteria (VMM) and viral lytic pressure (VLyP)

Viral mediated mortality of bacteria (VMM) was calculated as the ratio between VP estimate and the burst size (as determined by transmission electron microscopy). The fraction of bacterial production lysed by viruses per day was estimated from the bacterial production (cells/L/d) and viral mediated mortality (Helton et al., 2005; Winget et al., 2005). In addition, we calculated the ratio of VP to BP as an index of the viral lytic pressure (VLyP) as proposed by Motegi et al. (2009).

### Transmission electronic microscopy (TEM) analysis

Viral lytic infection was determined from the percentage of visibly infected cells (VIC) as previously described (Sime-Ngando et al., 1996). Bacterial cells were harvested by ultracentrifugation onto 400 mesh NI electron microscope grids with carbon-coated Formvar film using a Beckman Coulter SW40 Ti Swing-Out-Rotor run at 70,000 × g for 20 min at 4°C. Each grid was stained for 30 s with uranyl acetate (2% *w/w*), rinsed with 0.02 μm filtered distilled water to remove excess stain and dried on filter paper. Grids were examined using a JEOL 1200E × TEM operated at 80 kV at a magnification of 20,000–60,000 X to distinguish bacterial cells with and without intracellular viruses. At least 400–600 bacterial cells were inspected per grid to determine the percentage of VIC. For each sample, the burst size (number of viruses bacteria^−1^) was estimated from infected cells that were completely filled with viruses. Because mature phages are visible only late in the infection cycle, VIC counts were converted to the percentage of infected cells (% IC) using the equation % IC = 9.524 × VIC-3.256 (Weinbauer et al., 2002). The % IC was then converted to viral-induced bacterial mortality (VIBM, as a percentage of prokaryotic production) according to Binder (1999) using the equation VIBM = (% IC + 0.6 × % IC^2^)/(1–1.2 × % IC).

### Statistical analysis

A three-way ANOVA was used to understand the variations in biological parameters with respect to stations, seasons, depth, and time. Potential relationships among biological and environmental variables were tested by Karl Pearsons Correlation Analysis. Cluster/SIMPROF and non-metric multi-dimensional scaling (NMDS) was performed (using PRIMER 6 software) for segregating or spatial grouping of physico-chemical parameters during different seasons after normalizing the data. Clustering was carried out by Euclidean distance matrix using the group average method. The results obtained in dendrograms and NMDS were overlaid with biological parameters. Principal Component Analysis (PCA) was performed using PAST software (version 3) to understand the relationship between the biotic and abiotic variables. Distance based linear modeling (DistLM) analysis/distance based redundancy analysis (dbRDA) was performed for multivariate multiple regression analysis using Primer 7 software, to determine the relative importance of predictor variables (McArdle and Anderson, 2001).

## Results

During MON, the estuary was essentially a freshwater ecosystem due to heavy rains and riverine discharge. The salinity was low (<18) even at the inlets (S1 and S3, av. 8.6), while the rest of the stations were freshwater-dominated (S2, S4, and S5, av. 1.1; Supplementary Figure 1). The tidal fluctuations were more pronounced during PRM with high saline waters at the inlets and mesohaline waters upstream. The average tidal height in the inlets was 0.7 m, which decreased farther upstream (S4 and S5; 0.5 m). The salinity in the estuary increased to 29.7 ± 2.8 at S1 and to 9.9 ± 0.2 at S5 (Supplementary Figure 1). The highest/lowest salinity coincided with the highest/lowest tidal amplitude at all the locations. Salinity showed significant (*p* < 0.05) spatial and tidal variations, but diel variations were minimal (Supplementary Figure 1, Table 1). Throughout the study period, the water column was generally well-oxygenated, especially toward the freshwater zone (mean ± *SD* = 7.7 ± 0.4 mg L^−1^), where the concentrations of nutrients such as nitrate and silicate remained high. DO was high in the mesohaline regions (S2 and S4) and low at the inlets (Supplementary Figure 1). In contrast, PO_4_ concentration was high near the inlets and low farther upstream (S4 and S5). Concentrations of NO_3_, PO_4_, and SiO_4_ decreased considerably during PRM, and tidal variations were less pronounced (Table 1). Chl *a* was high throughout the estuary and ranged from 1.10 to 35.0 mg m^−3^. The highest values were observed in the mesohaline regions (mean ± *SD* = 25.0 ± 8.4 mg m^−3^ at S4) during PRM. Overall, Chl *a* showed significant diel, tidal and spatial variations (*p* < 0.05; Table 1).

**Table 1:** **Table showing results of the 3 WAY ANOVA for comparing spring and neap observations between different stations and between different time points**.

	**Parameters**	**Spring/Neap (A)**	**Stations (B)**	**Time (C)**	**A × B**	**A × C**	**B × C**
df		1	4	8	4	8	32
MON	Sal	15.525^**^	24.781^**^	1.878	10.047^**^	0.896	2.381^*^
	pH	24.059^**^	7.961^**^	1.228	15.370^**^	1.105	1.454
	NO_3_	0.688	19.234^**^	0.581	15.785^**^	0.662	1.099
	NH_4_	7.301^*^	7.763^**^	2.579^*^	1.524	1.883^*^	1.803
	PO_4_	1.38	26.666^**^	1.404	1.263	0.816	2.618^*^
	SiO_4_	0.029	4.176^**^	3.892^**^	4.919^**^	1.268	1.543
	DO	0.497	26.605^**^	2.652^*^	2.451	1.48	1.034
	BOD	15.785^**^	2.815^*^	3.226^**^	4.227^**^	0.804	2.138
	PA	13.47^**^	2.11	0.79	1.22	3.14^**^	1.26
	VA	0.99	9.23^**^	2.09	1.95	5.89^**^	1.63
	TVC	16.58^**^	1.71	1.48	0.27	1.67	1.28
	VP	4.89^*^	0.71	1.58	1.93	0.49	0.33
	BP	6.35^*^	27.91^**^	3.17^**^	8.29^**^	1.2	1.82^*^
	VPR	10.89^**^	5.24^**^	1.49	2.46^*^	1.4	1.57
	Chl *a*	23.08^**^	59.85^**^	2.98^*^	13.02^**^	1.19	1.92
	VTT	2.43	1.79	1.39	1.99	1.26	0.82
	% VMM	1,32	4,32	8,32	4,32	32,32	8,32
	VLyP	1.052	4.359^**^	1.378	6.683^**^	0.677	0.615
	%PA Lysed	0.042	0.835	1.113	2.321	0.588	0.485
	%BP Lysed	1.047	4.360^**^	1.377	6.677^**^	0.677	0.614
	C-Released	4.89^*^	0.7	1.58	1.93	0.49	0.33
PRM	Sal	2.387	127.550^**^	1.392	0.562	0.612	2.029
	pH	0.467	27.531^**^	1.588	2.136	0.506	1.442
	NO_3_	1.448	3.514^*^	3.160^**^	3.424^*^	1.136	0.646
	NH_4_	0.039	0.102	1.602	0.83	1.015	2.132
	PO_4_	0.702	26.099^**^	0.67	0.191	0.759	1.467
	SiO_4_	0.047	12.835^**^	4.690^**^	0.521	0.996	1.287
	DO	18.261^**^	26.250^**^	3.464^**^	6.516^**^	1.127	0.587
	BOD	1.228	2.607	3.717^**^	1.105	0.726	1.567
	PA	0.04	0.96	5.94^**^	1.58	2.56^*^	0.79
	VA	0.02	1.58	4.52^**^	0.8	1.51	0.73
	TVC	5.67^*^	7.58^**^	1.39	0.59	0.67	1.13
	VP	10.51^**^	8.77^**^	0.64	19.34^**^	0.89	0.69
	BP	6.41^*^	19.17^**^	2.49^*^	19.01^**^	1.78	1.91^*^
	VPR	2.32	1.39	0.92	1.3	1.17	1.34
	Chl *a*	0.22	27.19^**^	8.87^**^	1.65	0.63	4.33^**^
	VTT	5.92^*^	1.35	1.05	2.69^*^	1.13	0.91
	% VMM	10.509^**^	8.769^**^	0.636	19.334^**^	0.688	0.894
	VLyP	8.837^**^	12.018^**^	0.911	20.423^**^	1.094	1.199
	%PA Lysed	4.962^*^	6.232^**^	1.255	6.398^**^	0.998	1.406
	%BP Lysed	8.863^**^	12.011^**^	0.911	20.444^**^	1.095	1.2
	C-Released	10.51^**^	8.77^**^	0.64	19.34^**^	0.89	0.69

*Sal, salinity; NO_3_, nitrate; NH_4_, ammonia; PO_4_, phosphate; SiO_4_, silicate; DO, dissolved oxygen; BOD, biological oxygen demand; PA, prokaryotic abundance; VA, viral abundance; TVC, Viable Bacterial count; VP, viral production; BP, Bacterial Production; VPR, virus to prokaryote Ratio; Chl a, Chlorophyll a; VTT, Viral turn over time; % VMM, Viral mediated mortality; VLyP, Viral Lytic Pressure, % of PA lysed and % of BP lysed; C- Released, Carbon released*.

* Indicates significant F-values at p < 0.05 and

** *p < 0.01. Bold numbers implies significant values*.

### Variations in viral abundance, prokaryotic abundance, and viral-to-prokaryote ratio

Diel variations in the abundances of prokaryotes and viruses were measured over four tidal cycles at 3 h intervals (for 24 h) in two different seasons (monsoon and pre-monsoon) at five stations along the salinity gradient in the CE. In general, two high abundance peaks were noticed (one each during the day and night) with highest abundance during the day, especially during PRM. VA was high in PRM (av. 1.37 ± 0.71 × 10^7^ VLPs mL^−1^) when compared to MON (av. 1.13 ± 0.52 × 10^7^ VLPs mL^−1^). Viral abundance (VA) showed significant temporal variations and spatial variations (*p* < 0.001; Figures 2A, 3A, Table 1). PA was high (range = 0.24–4.2 × 10^6^ cells mL^−1^) in the estuary and showed significant variations (*p* < 0.001) with time and space. However, there were no significant tidal variations in VA and PA between two sampling days within the same season. The VA were in concurrence with PA (Figures 2A, 3A), which resulted in a strong correlation (*r* = 0.63, *p* < 0.05) between the two variables. VA was also correlated with salinity (*r* = 0.21, *p* < 0.05), TVC (*r* = 0.25, *p* < 0.05) and Chl *a* (*r* = 0.20, *p* < 0.05).

**Figure 2A:**
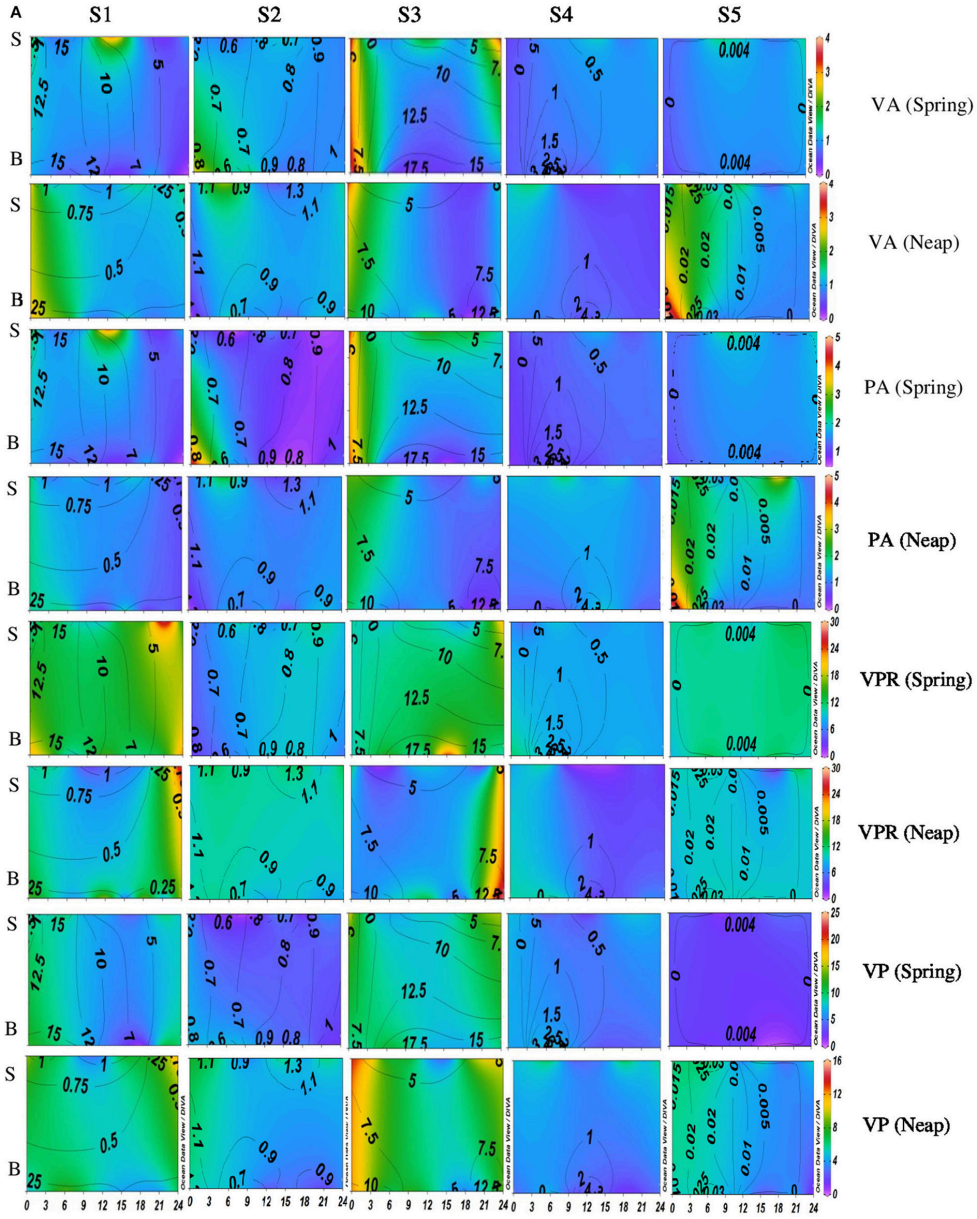
**Temporal variations (diel and tidal) in biological parameters during monsoon (MON) in CE**. X- axis represents the time series observations from 00:00 to 24:00 h and Y-axis represent the vertical section of the water column (S, Surface; B, Bottom). Z-axis represent different parameters in color contours and salinity is superimposed as the line contours. The vertical panels S1, S2, S3, S4, and S5 represents the five stations. The horizontal panels represent various parameters during MON, such as viral abundance (VA) (10^6^ VLPs mL^−1^), prokaryotic abundance (PA) (10^6^ Cells mL^−1^), virus-to-prokaryote ratio (VPR), and viral production (VP) (10^10^ VLPs L^−1^day^−1^), during spring and neap phases separately.

**Figure 2B:**
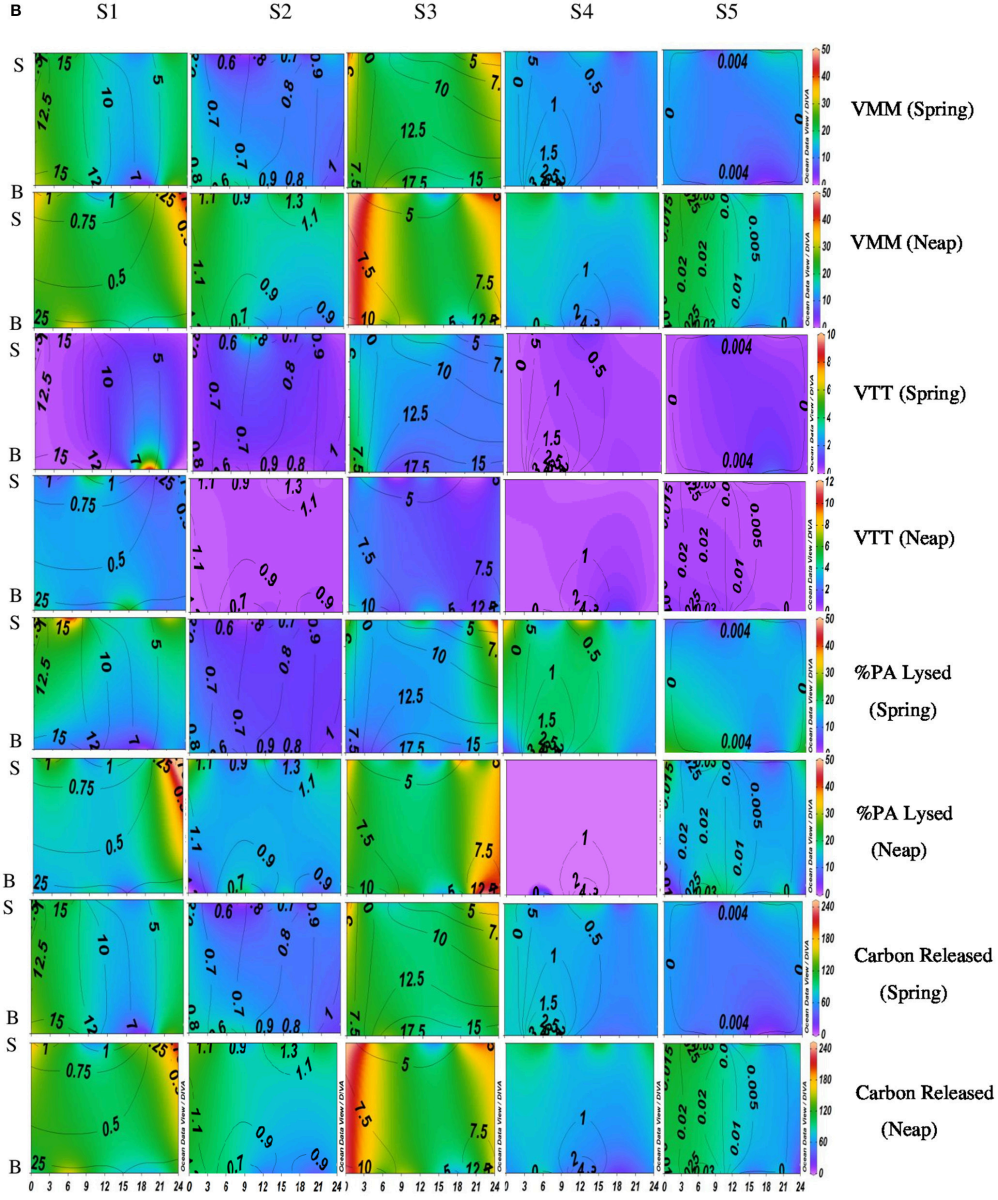
**Temporal variations (diel and tidal) in biological parameters during monsoon (MON) in CE**. X- axis represents the time series observations from 00:00 to 24:00 h and Y-axis represent the vertical section of the water column (S, Surface; B, Bottom). Z-axis represent different parameters in color contours and salinity is superimposed as the line contours. The vertical panels S1, S2, S3, S4, and S5 represents the five stations. The horizontal panels represent various parameters during MON, such as, Viral-mediated mortality (VMM%), Viral turnover time (VTT) (d^−1^), % of BA lysed, and Carbon released (μg/C/L/day) during spring and neap phases separately.

**Figure 3A:**
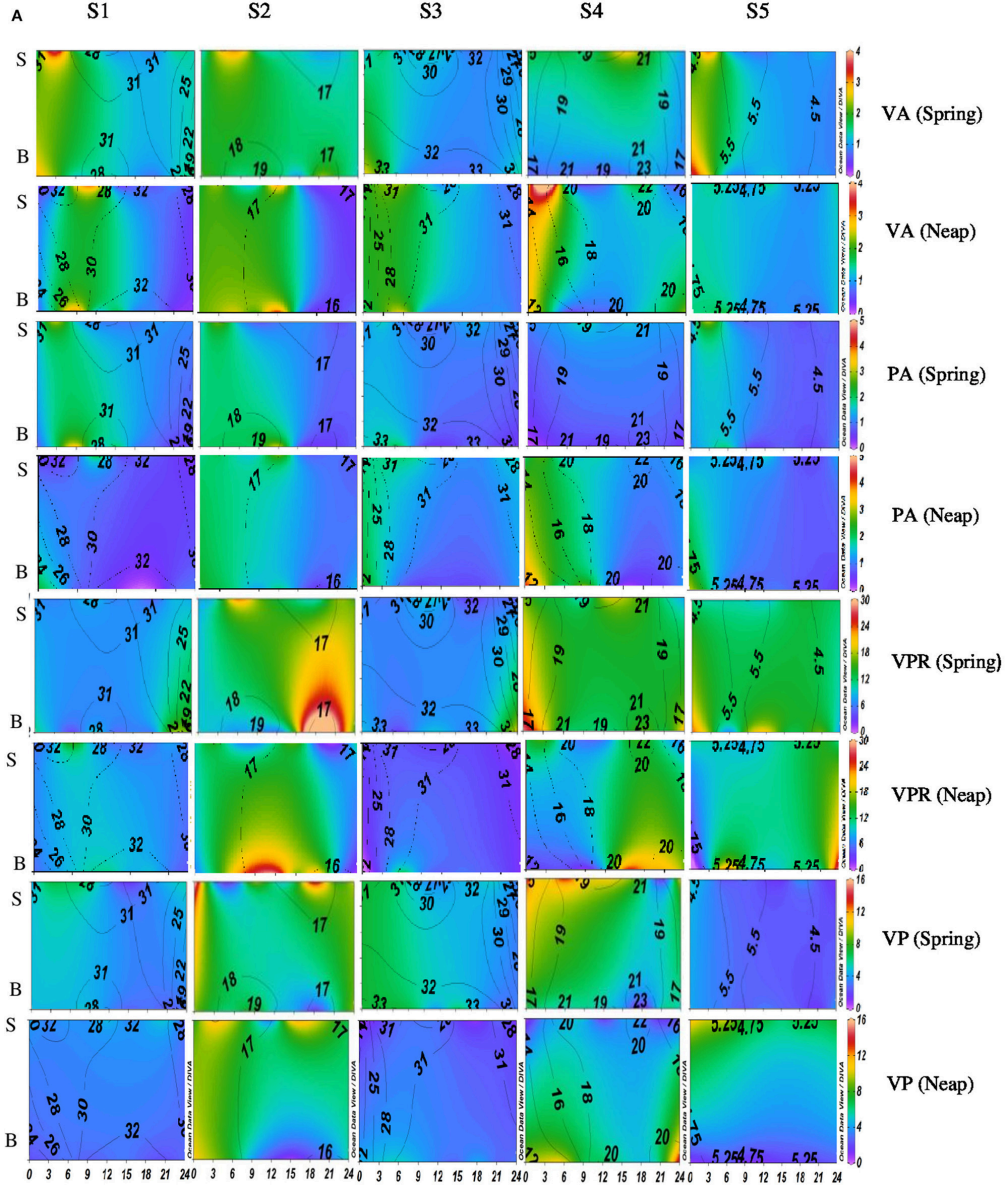
**Temporal variations (diel and tidal) in biological parameters during pre-monsoon (PRM) in CE**. X- axis represents the time series observations from 00:00 to 24:00 h and Y-axis represent the vertical section of the water column (S, Surface; B, Bottom). Z-axis represent different parameters in color contours and salinity is superimposed as the line contours. The vertical panels S1, S2, S3, S4, and S5 represents the five stations. The horizontal panels represent various parameters during PRM, such as viral abundance (VA) (10^6^ VLPs mL^−1^), prokaryotic abundance (PA) (10^6^ Cells mL^−1^), virus-to-prokaryote ratio (VPR), and viral production (VP) (10^10^ VLPs L^−1^day^−1^), during spring and neap phases separately.

The total viable bacteria (TVC) ranged from 0.65 to 13.30 × 10^5^ cells mL^−1^ with no significant diel variations (Table 1). TVC exhibited significant tidal variation (*p* < 0.001) at inlets, which was less pronounced toward upstream (S4 and S5). However, significant spatial and seasonal variations (*p* < 0.001) were seen in the viability of bacteria (Table 1). The mean TVC was higher in PRM (5.31 ± 2.69 × 10^5^ cells mL^−1^) when compared to MON (3.42 ± 1.45 × 10^5^ cells mL^−1^). The virus- to-prokaryote ratio (5–30) did not show any diel, tidal or seasonal variations during PRM, but tidal and seasonal variations were significant during MON.

### Variations in viral production (VP)

Significant variations (*p* < 0.001) in VP rates were observed over diel and tidal cycles during both MON and PRM (Table 1) with the higher rates during the day (Figures 2A, 3A). VP peaks were seen during the late morning and early evening hours (09:00–15:00 h of the day) during PRM, whereas during MON, the VP peaks were observed during early morning to noon hours (06:00–12:00 h). There were two high VP peaks during MON, whereas during PRM, many VP peaks indicated rapid viral replication and release. There were significant variations (*p* < 0.001) in VP rates during the spring and neap phases in both the seasons with higher rates during the neap phase (Figures 2A, 3A, Table 1). In general, VP rates were higher, especially in the mesohaline regions (S2 and S4) during PRM. VP was maximum at S4 (16.09 ± 2.42 × 10^10^ VLPs L^−1^ d^−1^) during PRM and minimum at S1 (4.20 ± 1.60 × 10^10^ VLPs L^−1^ d^−1^). The mean VP varied significantly (*p* < 0.001) with higher rates in PRM (9.56 ± 10.27 × 10^10^ VLPs L^−1^ d^−1^) than in MON (4.69 ± 2.44 × 10^10^ VLPs L^−1^ d^−1^).

### Variations in bacterial production, respiration, and bacterial growth efficiency

The CE sustained a high bacterial production (range = 9.78–87.01 μgC L^−1^d^−1^) with a mean BP of 36.66 ± 15.50 μgC L^−1^d^−1^ (**Figure 4**). However, there were no significant diel variations in BP rates (Table 1), but there was a steep decline in BP during the day, probably due to bacterial decay. BP was significantly (*p* < 0.05) high during spring phase compared to the neap phase of the tide. Spatial variations in BP were significant (*p* < 0.001) with high saline regions showing maximum rates. Seasonally, BP rates were significantly (*p* < 0.001) higher during PRM (av. 41.81 ± 18.35 μgC L^−1^d^−1^) when compared to MON (av. 37.30 ± 11.84 μgCL^−1^d^−1^).

The bacterial respiration (BR) was found to be higher than the bacterial production in CE. Mean bacterial respiration (BR) was 66.6 ± 51.8 μg C L^−1^d^−1^ in surface waters (**Figure 4**). Notably, BR at S4 was consistently high during PRM and in S1 at MON seasons. BP and BR were assessed to determine variations in BGE, which we used as an index of prokaryotic metabolism at the community level. BGE in CE was consistently high indicating that the system is productive. The BGE showed significant spatial and seasonal variations (*p* < 0.05), which was maximum during PRM (mean ± *SD* = 55.28 ± 23.08%), especially at the inlets. BGE was minimum at the mesohaline regions, especially at S4, indicating that a higher flux of carbon is being respired rather than converted into biomass in the mesohaline region (**Figure 4**).

### Viral-mediated mortality, viral turn over time, and viral lytic pressure

VMM in CE was generally high during daytime and varied from 0.46 to 55.3% (Figures 2B, 3B). There was significant variation (*p* < 0.001) in VMM with time and tide. Seasonally, VMM rates were higher (20.02 ± 11.34) during PRM especially in the mesohaline regions (S2 and S4**;** Supplementary Table 2). However, during MON, VMM was comparatively high (11.72 ± 6.73) at the inlets (Supplementary Table 1). Viral turnover time (VTT) ranged from 0.05 to 12.83 d^−1^ (Figures 2B, 3B). Though VTT was high in the daytime, there were no significant diel, tidal, and spatial variations during MON. However, the rates were higher during PRM with significant tidal and spatial variability (*p* < 0.001; Figures 3B, 4B, Table 1). Viral lytic pressure (VLyP) ranged from 0.08 to 22.08 during the study period. Although, there were significant spatial variations (*p* < 0.001) in VLyP, diel, and tidal variations were not significant during MON (Table 1). During PRM, the VLyP were higher with significant tidal and spatial variations (*p* < 0.001; Supplementary Table 2).

**Figure 3B:**
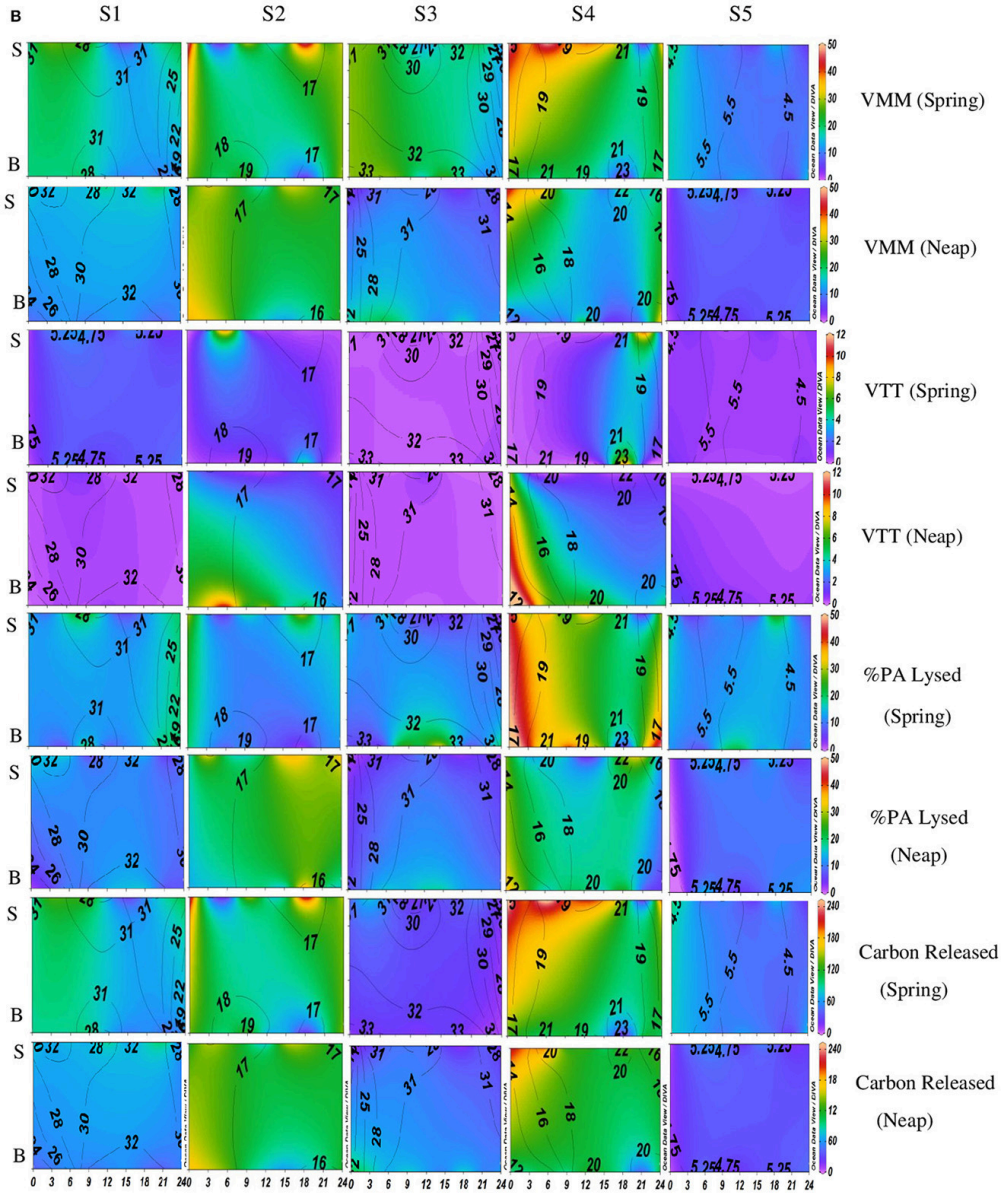
**Temporal variations (diel and tidal) in biological parameters during pre-monsoon (PRM) in CE**. X-axis represents the time series observations from 00:00 to 24:00 h and Y-axis represent the vertical section of the water column (S, Surface; B, Bottom). Z-axis represent different parameters in color contours and salinity is superimposed as the line contours. The vertical panels S1, S2, S3, S4, and S5 represents the five stations. The horizontal panels represent various parameters during PRM, such as, Viral-mediated mortality (VMM%), Viral turnover time (VTT) (d^−1^), % of BA lysed, and Carbon released (μg/C/L/day) during spring and neap phases separately.

**Figure 4:**
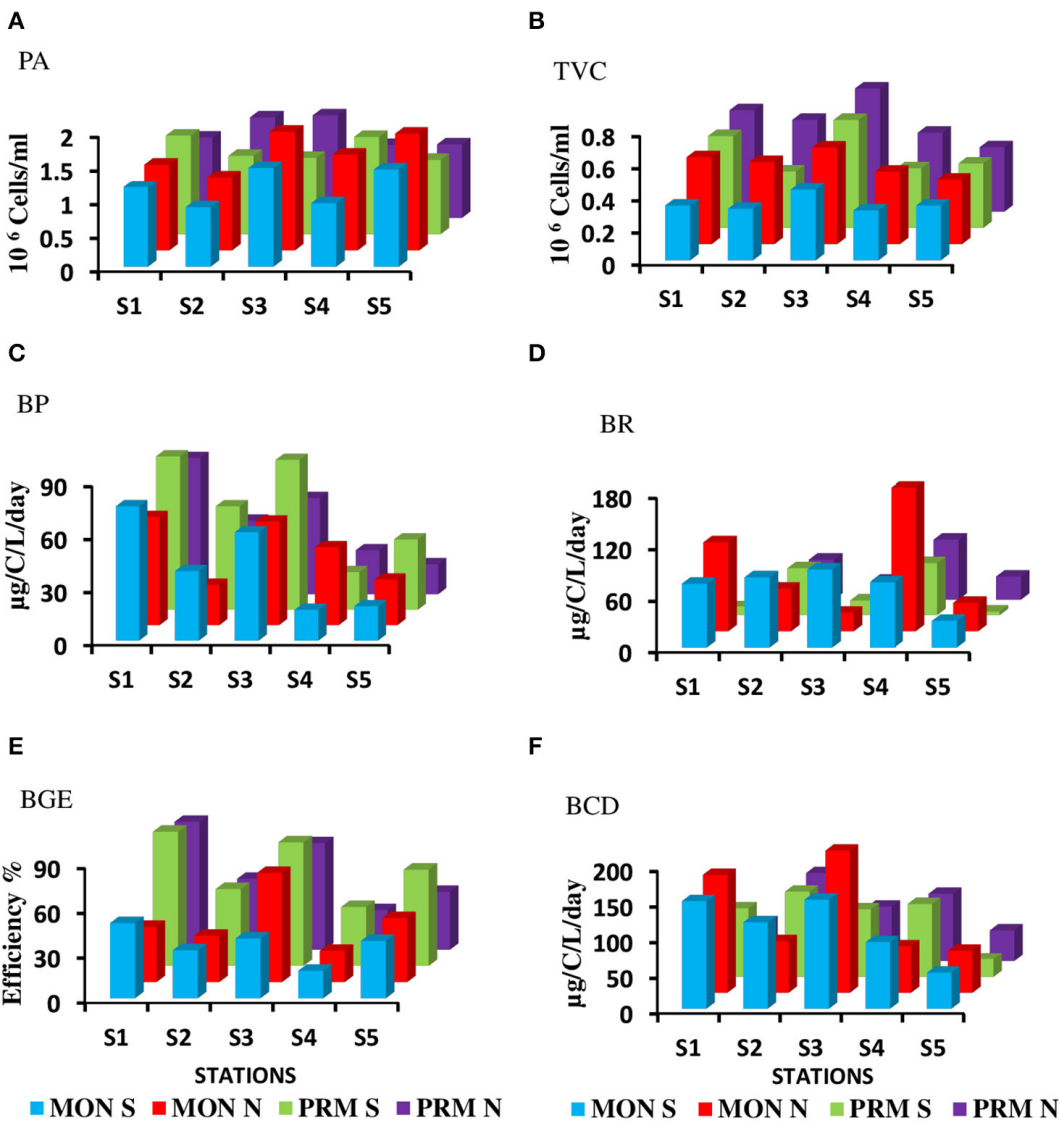
**Mean distribution of bacterial parameters during monsoon (MON) and Pre-monsoon (PRM) (A)** Prokaryotic abundance in 10^6^ cells/ml (PA) **(B)** Viable bacterial abundance in 10^6^ cells/ml (TVC) **(C)** Bacterial production in μg/C/L/day) (BP) **(D)** Bacterial respiration in μg/c/l/day) (BR), **(E)** Bacterial growth efficiency % (BGE) **(F)** Bacterial carbon demand in (μg/c/l/day) (BCD). Spring and neap tide observations are denoted as S and N, respectively.

The rate of lysis of prokaryotic cells, calculated from PA and BP, showed significant variation (*p* < 0.001) with time and tide. There was no specific pattern for the lysis rates during a diel cycle, but the lysis rates were higher during PRM (80.25%) compared to MON (25%; Figures 3B, 4B). Interestingly, the maximum lysis rates were higher in the mesohaline regions during both the seasons (Supplementary Tables 1, 2). The lysis rates were 33.84 ± 15.97 at S4 during PRM and at S3 (28.71 ± 16.20) during MON where the mesohaline conditions prevailed. The carbon released through viral mediated bacterial lysis showed significant seasonal and spatial variations (*p* < 0.001). The rates ranged from 2.24 to 265.44 μCL^−1^d^−1^. The rates were higher during PRM when compared to MON. The amount of carbon released due to viral-mediated mortality was high at S4 during PRM (142.90 ± 77.81) and at S3 (117.2 ± 37.24) during MON. TEM results also supported the observation that VIBM was significantly higher (*p* < 0.001) in the mesohaline regions (S2 and S4) during PRM (Table 2).

**Table 2:** **Table showing “Viral lytic infection and burst size” estimates by transmission electron microscopy (TEM) observations**.

	**VIC%**	**%IC**	**VIBM%**	**BS mean (min-max)**
**MONSOON**				
S1	1.48	10.84 ± 2.2	13.00	27 (11–41)
S2	1.10	7.22 ± 1.8	8.11	16 (12–19)
S3	1.51	11.12 ± 1.9	13.42	47 (19–92)
S4	1.10	7.22 ± 1.8	8.16	22 (14–32)
S5	1.20	8.17 ± 1.5	9.3	25 (16–31)
**PRE MONSOON**				
S1	1.21	8.27 ± 1.5	9.44	14 (08–109)
S2	1.99	15.7 ± 2.6	20.73	20 (10–32)
S3	1.76	13.51 ± 2.5	17.1	41 (06–109)
S4	1.9	14.84 ± 2.1	19.27	25 (12–36)
S5	1.44	10.46 ± 2.5	12.47	22 (12–35)

*VIC, Frequency of visibly infected bacterial cells; % IC, percentage of infected bacterial cells; VIBM, Viral mediated bacterial mortality; BS mean, Burst size mean (minimum-maximum)*.

**Figure 5:**
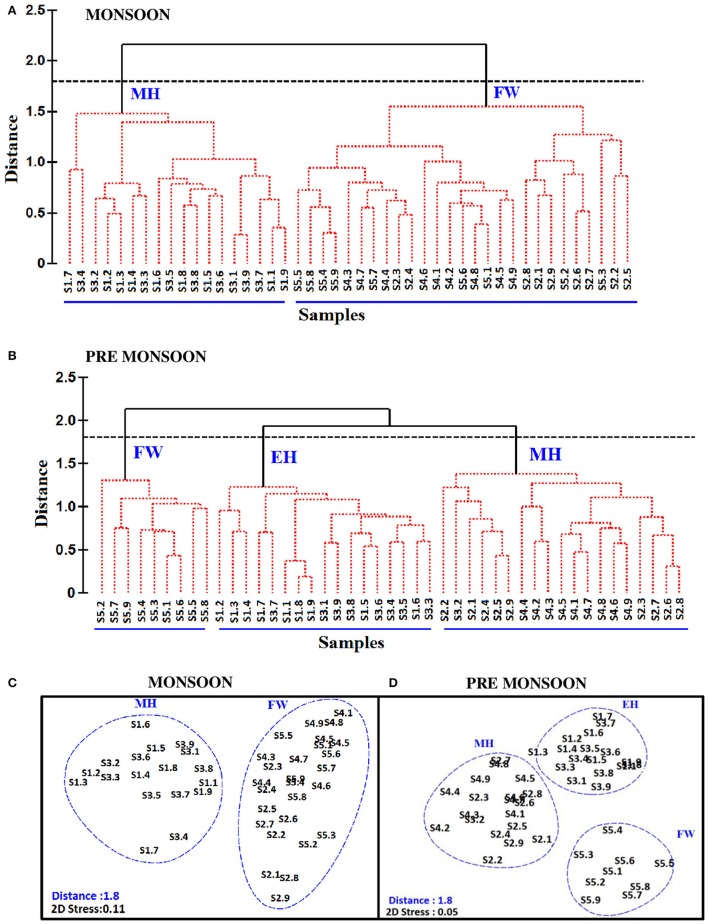
**Cluster plot representing the segregation of stations based on the distribution of physico-chemical and biological parameters during (A)** monsoon (MON) and **(B)** pre-monsoon (PRM). The stations are represented from S1 to S5. Numbers 1-9 represent the nine time series observations carried out at each sampling station (i.e: S1.1, S1.2,…to S1.9 represent time series observations at station S1). Non metric multidimensional scaling (NMDS) is shown in panels **(C,D)**, which represent the distribution of time point observations at different stations using salinity and biological parameters during MON and PRM, respectively. The clusters are named as euryhaline (EH), mesohaline (MH) and freshwater (FW), based on the salinity values.

**Figure 6:**
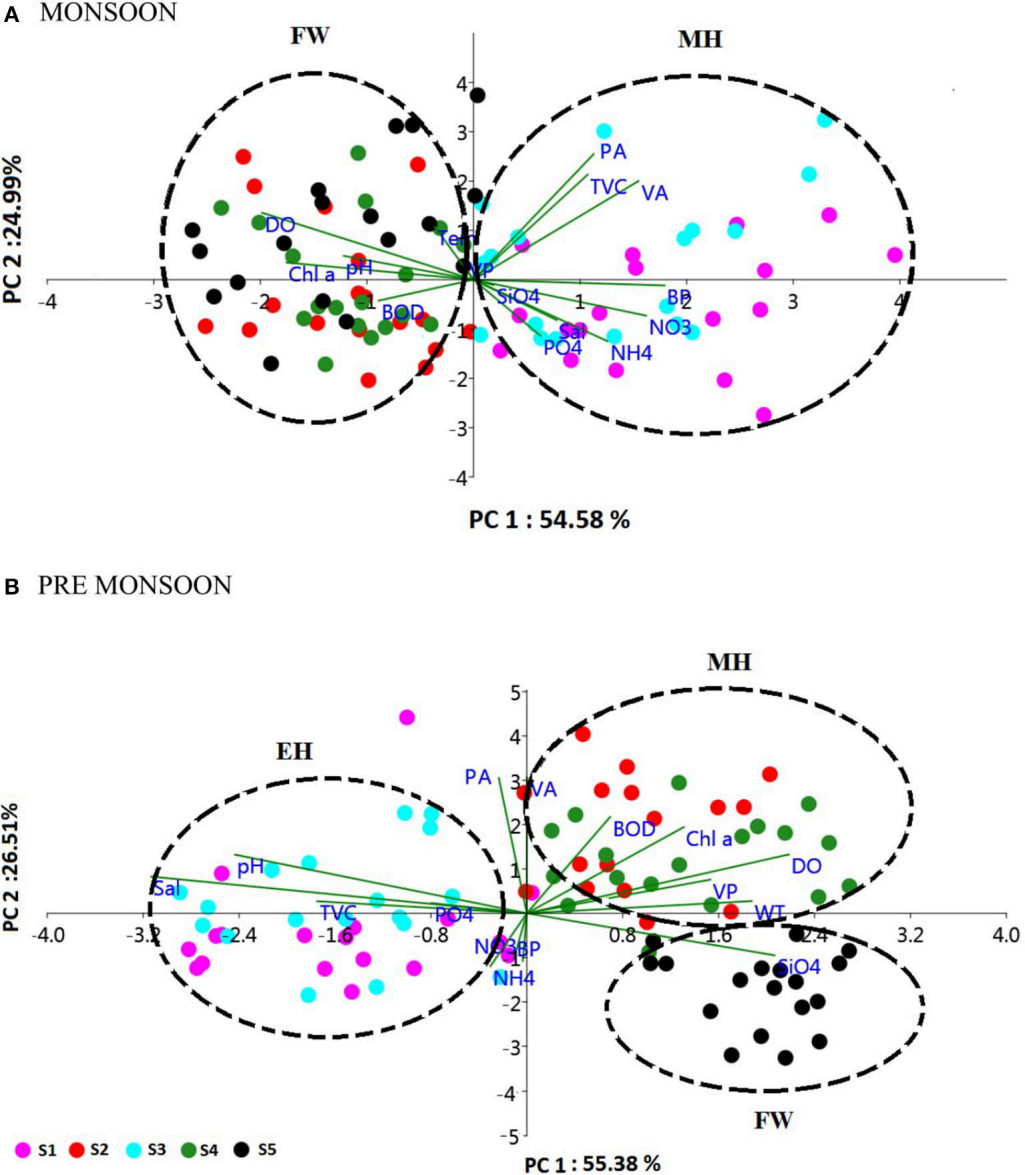
**Principal component analysis (PCA) biplot representing the distribution of biological parameters and interrelationships of physico-chemical and biological parameters during (A)** monsoon (MON) and **(B)** pre-monsoon (PRM). The sampling stations (S1–S5) are represented as different color spots. The % variance explained by PC1 and PC2 is 54.58 and 24.99% in MON and 55.38 and 26.51% in PRM, respectively. The clusters are named as euryhaline (EH), mesohaline (MH) and freshwater (FW), based on the salinity values.

**Figure 7:**
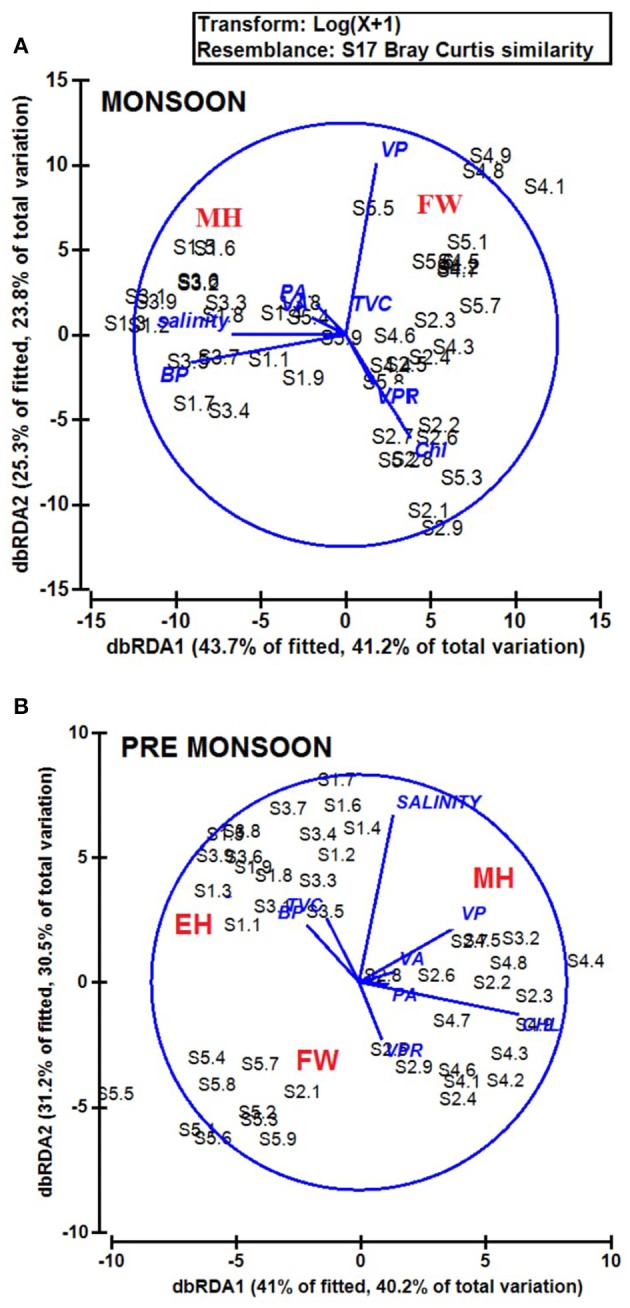
**Distance based linear model (DistLM) or distance based redundancy analysis (dbRDA) plot showing the interrelationships of physico-chemical and biological parameters during (A)** monsoon (MON) and **(B)** pre-monsoon (PRM). The fitted model explained 52.7% of the total variation during monsoon and 40.5% variation during pre-monsoon. The % variance explained by dbRDA1 and dbRDA2 is 56.7 and 19.7% in MON and 48.6 and 29.6% in PRM, respectively.

The biological parameters in the mesohaline regions were distinct compared to the other saline zones of the estuary. The cluster and NMDS analysis could indicate distinct patterns in the distribution of physicochemical parameters. The cluster and NMDS analysis categorized the study region into three saline zones during PRM, namely a high saline zone (>30) composed of two stations (S1 and S3), a medium saline zone (S2 and S4) and a freshwater zone (S5) with salinity <5 during PRM. However, during monsoon, only two zones were distinct, <18 (S1 and S3) and freshwater zone (S2, S4, and S5). The biological parameters also followed the same pattern and were overlaid onto the NMDS plots (Figure 5). The PCA was used to determine the relationship between the environmental and biological variables (Figure 6). PCA indicated that PA was the best predictor variable for VA during PRM and both PA and TVC during MON. PCA also clearly indicated the zonal preference of bacteria and viruses for mesohaline regions during both seasons, which corroborated well with the cluster and NMDS analysis. DistLM analysis indicated that viral dynamics is mostly host dependent with more activity is the mesohaline regions (Figure 7). The different salinity zones were clearly seen in the DistLM analysis. The variables such as PA, TVC, BP, VP, Chl *a*, and salinity (*p* < 0.05) accounted for 72–73% of overall variance in viral abundance (Table 3) using non-parametric multivariate regression.

**Table 3:** **Showing distance based linear model (DistLM) on multivariate regression analysis for viral abundance**.

**Variable**	**F^b^**	**P^c^**	**CumL^d^ (%)**
**MONSOON**			
PA	2.86	3.99	59.43
BP	5.41	38.87	93.09
TVC	0.59	2.09	85.58
Salinity	33.314	36.66	46.02
VP	10.17	34.19	83.97
Chl *a*	7.58	13.93	69.91
**PRE MONSOON**			
PA	1.04	0.86	36.15
TVC	5.88	5.42	43.77
Salinity	17.24	12.36	22.32
Chl *a*	11.32	13.75	58.43
VP	11.42	29.47	81.43
BP	6.34	9.35	66.64

*PA, Prokaryotic abundance; BP, bacterial production; TVC, Viable Bacterial count; Salinity; VP, viral production; Chl^a^, chlorophyll a, accounted for 72–73% of the overall variance in both seasons. These factors significantly (p < 0.05) correlated with the variances in viral abundance. F^b^, Pseudo F-values; P^c^, P-values; CumL^d^, cumulative percentage of variance explained*.

## Discussion

This study provides a new extensive dataset to explore environmental factors affecting the distribution of viruses and viral activity in a complex tropical estuarine system (Cochin estuary) impacted by strong annual monsoonal cycles. CE is the largest monsoonal estuary along the west coast of India which receives freshwater inflow from six rivers and salinity incursion from the adjoining Arabian Sea. As the rivers bring enormous amounts of freshwater during the peak MON (July–August), the CE transforms into a freshwater system except near the inlet region. On the other hand, during the PRM (March–May), the low freshwater inflow of rivers allows active salinity incursion and, as a result, the downstream portion of the CE transforms into an extension of the adjacent Arabian Sea (Jyothibabu et al., 2006). The present investigation carried out in spatial and seasonal scale over a diel cycle during the pre-monsoon (dry) and monsoon (wet) seasons, revealed that viral distribution and activity were regulated by host abundance (mainly bacteria) and activity. Physico-chemical parameters, like salinity, regulate the distribution of host organisms, which in turn influence the viral distribution and activity. Among the salinity regimes, the mesohaline region of the estuary was found to be the preferred zone supporting high viral abundance, lysis, and production due to high bacterial activity. PCA indicated that host abundance (PA and TVC) was the best predictor for VA. Cluster, NMDS, and DistLM analysis showed the existence of three distinct zones in the estuary based on variation in salinity during PRM and two zones during MON (Figures 5, 7). The biplots (Figure 6), derived from the PCA analysis further depict the influence of environmental parameters on the distribution and activity of viruses and bacteria with seasons. PCA plots revealed that VA and PA showed closeness to mesohaline quadrants during both the seasons indicating their preference for mesohaline conditions (Figure 6). Most important predictor variables for viral abundance were PA, TVC, BP, VP, Chl *a*, and salinity (*p* < 0.05) accounting for 72–73% of overall variance in viral abundance (Table 3).

The viral abundance and production rates measured during the present study were comparable with previous reports from other estuarine and marine systems (Simek et al., 2001; Jiao et al., 2006; Weinbauer et al., 2007; Bettarel et al., 2011; Winget et al., 2011). Viral abundance and production are generally known to increase with trophic status since eutrophic conditions have the capacity to support larger and more active host communities (Helton et al., 2006; Li et al., 2010). In both PRM and MON seasons, prokaryotes were the major hosts for viruses, where variation in viral abundance was predicted by prokaryotic abundance alone (Figures 6, 7). A study by Parvathi et al. (2013) on CE has attributed short-term variations in VP to various host processes and environmental factors, including light and salinity. The above findings are corroborated in this study with significant correlation (*p* < 0.001) of VA with PA and BP, as previously reported for different environments (Parvathi et al., 2011; Winget et al., 2011; Payet and Suttle, 2013; Pradeep Ram and Sime-Ngando, 2014). The host parameters were influenced by seasonal salinity variations, which in turn triggered the spatial and seasonal variations in viral parameters. Salinity is reported to be the second most important predictor factor (after bacterial abundance) for viral abundance on a spatial scale and temperature on a temporal scale (Auguet et al., 2005). However, deil variations in VA and VP could be host dependent or due to solar/UV radiation instead of the tidal effects. VP was found to be higher during the day and its decrease in the afternoons could be due to the degradation of viruses, loss of infectivity, or preferential infection of host cells to avoid UV damage (Winget and Wommack, 2009). Previous studies have shown that viruses are extremely sensitive to solar radiation (Suttle and Chen, 1992; Wommack et al., 1996; Noble and Fuhrman, 1997; Wilhelm et al., 1998). VP and % BA lysed were high during the night presumably due to the release of viral particles from host cells when the risk of UV damage is minimum. As viruses are reliant upon their hosts, diel patterns in VA or VP could be linked to diel or seasonal changes in host abundance, diversity, and activity, which are in turn influenced by environmental factors. These factors are not mutually exclusive but work in tandem to produce the observed patterns of diel and tidal fluctuations.

Interestingly, seasonal and spatial variability were conspicuous when compared to the short term diel or tidal variations in the present study. Viral abundance, lytic production, and their activity were high during PRM, especially in the mesohaline regions of the estuary. High infection rates in CE were comparable with those values reported from Chesapeake Bay (Wilhelm et al., 2002). In this estuarine system, higher viral activity in mesohaline regions could be due to several reasons, the most important being the seasonal variations in estuarine hydrography (salinity intrusion and precipitation). Interestingly, this estuary undergoes an extensive transformation from a well-mixed estuary with limited saltwater intrusion at the inlets during MON, to a highly stratified water body during PRM. Hence, the major hydrological variable is salinity. The salinity gradient drives the existence of diverse species which can propagate under oligohaline, mesohaline, or marine conditions (Sreedevi, 2007; Sooria et al., 2015). As viruses are dependent on the metabolic status of their hosts, factors such as salinity that affect host activity can critically influence viral proliferation.

The present study reports low BGE with higher respiratory losses in the mesohaline region, indicating that a higher flux of carbon is respired through bacteria than converted into biomass. The mesohaline regions of the estuary either lie between two inlets (such as S2) or between the inlet and the freshwater region/Vembanad Lake (such as S4). This makes these regions less dynamic with low flushing rates during the dry pre-monsoon season with no rainfall and less riverine influx. Moreover, due to the closure of Thannermukkam bund during PRM, the riverine influx from the Vembanad Lake (S5) is blocked as a safety measure to protect the paddy fields in the southern regions. This has changed the fresh and salt water balance of the estuary, resulting in reduced flushing out of organic pollutants from the estuary, especially from these less dynamic mesohaline regions. Organic pollutants are consumed by naturally occurring bacteria, which is reflected in the high bacterial respiration and low BGE. The contribution of viral lysis to the DOC pool was high during PRM (10.8 ± 2.8%) when compared to MON (5.5 ± 1.7%) with highest being in mesohaline regions. DOC was high throughout the study period with highest values during PRM (2.77 ± 0.87 gL^−1^) and MON (2.73 ± 2.33 gL^−1^). The role of bacteria as oxidizers of organic matter, hence as CO_2_ producers, and remineralizers of N, P, or Fe (Middelboe and Lyck, 2002; Bonilla Findji et al., 2009) is well-documented. CE, being a highly eutrophic ecosystem, inorganic nutrients, and DOC always remained high and never limited bacterial activities. However, spatially, salinity variations were significant (*p* < 0.001; Table 1). Studies carried out in the tropical Red river estuary (Northern Vietnam) have suggested that salinity levels impact bacterial respiration rates as determined from the percentage of CTC + cells (Marine et al., 2013). It has also been demonstrated that salinity alters the conformation and size of organic matter in estuaries (Baalousha et al., 2006). Moreover, increasing ionic strength tends to alter the association of organic matter with the inorganic matrix, which also can alter bioavailability (Keil et al., 1994). Therefore, higher bacterial respiration in the mesohaline zones indicates that the bioavailability of DOM increased bacterial metabolism in this region. Alternatively, salinity can also influence the viral proliferation independent of host growth by phage-specific effects on production. For example, the phage nt-1 of *Bacillus natriengens* showed longer latent periods and highly reduced burst sizes (plaque forming units) at salinities below 18, while nt-6 showed highest phage production rates at brackish salinities (below the host's growth optimum; Zachary, 1976). In estuaries, where the salinity is known to affect specific phylogenetic groups, marked changes in cell-specific activity and growth efficiency can be expected (del Giorgio and Bouvier, 2002). This exemplifies the salinity-induced virus–host interactions and suggests mechanisms for alterations in viral population dynamics under changing salinity in estuarine environments (Mojica and Brussaard, 2014). In the present study, the viral processes were more in the mesohaline regions when compared to the high saline regions. The high saline regions in the estuary were restricted to the inlets, which were highly dynamic due to tidal action. Shifts in salinity may alter the integrity of the capsid receptors and inhibit the binding of viruses to their hosts (Kukkaro and Bamford, 2009), which probably resulted in significant reduction in viral standing stock and infection at high salinity zones in our study. Alternatively, the inactivation and decay of viruses itself could be influenced by changes in ionic strength (Wells and Deming, 2006).

Host and viral community composition could be another reason for increased viral activity in the mesohaline zone as observed in this study. TEM results suggested that there was significant seasonal variation in the bacterial morphotypes infected with viruses. During monsoon, all the morphotypes, viz., cocci, short rods, long rods etc. were infected by viruses in CE, whereas during pre-monsoon only rods were found to be infected with the viruses (Pradeep Ram, personal communication). Increased input of allochthonous bacteria through freshwater inflow during MON than during PRM could have resulted in high host diversity in the former. Viral-host contact rates are more successful when the host diversity was low resulting in high viral production rates (Kan et al., 2006). Large changes in the composition of bacterial community across the salinity gradient have been previously reported in temperate and tropical estuarine systems (Bouvier and del Giorgio, 2002; Bettarel et al., 2011; Campbell et al., 2011). Even moderate changes in salinity have been shown to induce changes in prokaryote composition (Langenheder et al., 2003) and viral morphotypes (Middelboe and Lyck, 2002). The lysis of the high percentage of the bacterial community in mesohaline regions may also be due to the lack of resistance to co-occurring phages through increased contact rates between viruses and susceptible host cells.

Another reason for higher viral activity in mesohaline regions is the grazing pressure. Zooplankton biomass was high throughout the study area during PRM (av. 8.02 ± 0.96–11.7 ± 1.3 mg C m^−3^) when compared to MON (av. 0.24 ± 0.96–0.48 ± 0.5 mg C m^−3^). The abundance of microzooplankton grazers was noticeably high in the mesohaline regions during PRM (mean = 3.11 × 10^4^ l^−1^ at S4, against mean = 1.11 × 10^4^ l^−1^ at S3; part of this study, published by Sooria et al., 2015). High grazing pressure has been shown to enhance viral lysis and production (Weinbauer et al., 2007; Pradeep Ram and Sime-Ngando, 2008). The high abundance of grazers, especially during PRM in mesohaline regions of the estuary, suggests that grazers can serve as important causative agents of bacterial mortality, thus affecting the viral production, directly and/or indirectly (Sooria et al., 2015). In fact, during PRM, all the planktonic components, especially the grazers were much more abundant that in MON. Consequently, each interaction between the different prey-predator pairs occurred effectively. When grazers are present at high rates, virus-specific selective grazing (selective grazing on virus- infected host cells) has the potential to influence the specific virus–host dynamics and affect biodiversity (Berdjeb et al., 2011). Selective grazing of virally-infected host cells will alter the contact rate between virus and uninfected host cell by reducing the number of virus progeny released from the remaining infected host cells (Ruardij et al., 2005; Mojica and Brussaard, 2014). Interactions among viruses, bacteria, and grazers are complex, but due to the lack of experimental investigation, the synergistic effects of grazers on bacteria-virus interaction has to be further validated.

## Conclusion

This study has attempted to understand the short term (diel and tidal), seasonal and spatial variations in viral processes in a highly dynamic tropical monsoonal estuary in India. Viruses were the most abundant planktonic component of CE. The results suggest conspicuous seasonal and spatial variations in viral parameters in the ecosystem studied. Their dynamics was shaped greatly by prokaryotes and TVCs during the study period. In this estuarine system, apart from prokaryotic hosts, salinity patterns, directly or indirectly influenced the virioplankton distribution and viral infection on a spatial and seasonal scale. The mesohaline regions of the estuary act as hot spots for intense host activity where viral lytic infections can effectuate major changes in the microbial food web. The present study suggests that the viruses are key players in the microbial loop throughout the year, with spatial shifts in their activity dictated by host abundance, and medium saline conditions. Viral shunt thus exerts a strong influence on the processes occurring in the plankton food web and the related carbon and energy flows. However, further studies are necessary to understand the regulation of viral control on host physiology and metabolism and its impact on community composition in tropical systems.

## Author contributions

VJ and AP have collected and analyzed samples for viral parameters and preparation of the manuscript. ASP and TS are responsible for transmission electron microscopy and preparation of the manuscript. KKB and NVM have performed analysis of chemical parameters. CR has provided the physics data. NVM and RJ have helped in sample collection, analysis of biological samples and manuscript preparation. KVJ has performed statistical analysis of the data.

### Conflict of interest statement

The authors declare that the research was conducted in the absence of any commercial or financial relationships that could be construed as a potential conflict of interest.
