# Supplementary material for: Viral-Induced Mortality of Prokaryotes in a Tropical Monsoonal Estuary
Source: Front Microbiol. 2017 May 23;8:895. doi: 10.3389/fmicb.2017.00895 (PMC5440509; doi:10.3389/fmicb.2017.00895)
Supplement: Supplementary file 1 [file Table1.DOCX]

|  | **Parameters** | **S1** | **S2** | **S3** | **S4** | **S5** |
| --- | --- | --- | --- | --- | --- | --- |
| **MON(S)** | **VA** | 1.22 ± 0.38 | 1.04 ± 0.36 | 1.21 ± 0.55 | 0.86 ± 0.24 | 0.09 ± 0.27 |
|  | **PA** | 0.93 ±0.37 | 1.07 ± 0.66 | 1.07 ± 0.55 | 0.84 ± 0.27 | 0.89 ± 0.30 |
|  | **TVC** | 3.17 ±1.46 | 3.57 ± 1.46 | 3.80 ± 1.96 | 2.86 ± 1.02 | 3.20 ± 0.90 |
|  | **VPR** | 14.02 ±5.34 | 10.89 ± 2.73 | 12.50 ± 4.95 | 10.54 ± 1.70 | 11.14 ± 1.33 |
|  | **BP** | 76.37 ±42.01 | 39.27 ± 8.78 | 61.66 ± 21.79 | 17.37 ± 6.46 | 19.4 ± 14.58 |
|  | **VP** | 4.65 ±1.87 | 2.84 ± 1.58 | 6.11 ± 1.94 | 3.51 ± 1.10 | 2.71 ± 1.30 |
|  | **VMM** | 18.61 ±7.50 | 11.35 ± 6.32 | 24.42 ± 7.76 | 14.03 ± 4.40 | 10.84 ± 5.19 |
|  | **VTT** | 0.97 ± 2.96 | 0.83 ± 1.45 | 0.20 ± 0.08 | 0.30 ± 0.21 | 0.54 ± 0.51 |
|  | **VLyP** | 1.68 ± 1.24 | 1.46 ± 0.80 | 2.20 ± 1.07 | 4.51 ± 2.22 | 3.88 ± 2.30 |
|  | **%PA LYSED** | 22.40 ±11.70 | 12.39 ±7.82 | 28.71 ±16.20 | 18.02 ±7.82 | 13.18 ±7.41 |
|  | **%BPLYSED** | 6.72 ± 4.95 | 5.83 ± 3.18 | 8.81 ± 4.27 | 18.48 ± 9.80 | 15.51 ± 9.18 |
|  | **C-RELEASED** | 89.32 ±35.98 | 54.49 ± 30.35 | 117.2 ± 37.24 | 67.33 ± 21.1 | 52.0 ± 24.92 |
| **MON(N)** | **VA** | 1.39 ± 0.58 | 1.11 ± 0.49 | 1.52 ± 0.67 | 0.71 ± 0.38 | 1.23 ± 0.69 |
|  | **PA** | 1.22 ± 0.60 | 1.12 ± 0.47 | 1.44 ± 0.68 | 1.20 ± 0.43 | 1.46 ± 0.97 |
|  | **TVC** | 5.98 ± 3.07 | 4.70 ± 1.93 | 6.36 ± 3.21 | 4.24 ± 1.35 | 3.94 ± 1.66 |
|  | **VPR** | 12.39 ± 4.54 | 10.04 ± 1.75 | 11.53 ± 3.39 | 6.65 ± 3.66 | 9.44 ± 2.38 |
|  | **BP** | 61.62 ± 36.48 | 22.85 ± 10.89 | 59.00± 40.59 | 44.51 ± 25.2 | 41.8 ± 18.35 |
|  | **VP** | 6.57 ± 2.37 | 4.52 ± 1.62 | 7.81 ± 3.14 | 3.77 ± 1.76 | 4.43 ± 1.68 |
|  | **VMM** | 26.27 ± 9.47 | 18.08 ± 6.47 | 31.22 ± 12.56 | 15.06 ± 7.03 | 17.72 ± 6.73 |
|  | **VTT** | 3.09 ± 1.61 | 0.25 ± 0.12 | 1.85 ± 1.52 | 0.28 ± 0.38 | 0.29 ± 0.16 |
|  | **VLyP** | 6.34 ± 3.44 | 2.38 ± 1.43 | 7.52 ± 4.52 | 2.38 ± 1.78 | 2.54 ± 1.44 |
|  | **%PA LYSED** | 20.06 ± 14.06 | 14.83 ± 8.58 | 26.80 ± 12.76 | 13.61 ± 6.68 | 15.55 ± 8.45 |
|  | **% BP LYSED** | 24.35 ± 10.52 | 9.23 ± 5.96 | 31.17 ± 15.23 | 8.41 ± 5.24 | 10.16 ± 5.77 |
|  | **C-RELEASED** | 126.08 ± 45.4 | 86.77 ± 31.05 | 149.8 ± 60.28 | 72.3 ± 33.77 | 85.0 ± 32.32 |

**Supplementary Table:1: Table showing average ± standard deviation of various parameters at stations, S1, S2, S3, S4, and S5 during monsoon season, for spring [MON (S)] and neap [MON (N)] tides separately. Abbreviations used are viral abundance (VA), prokaryotic abundance (PA), Viable Bacterial count (TVC), virus to prokaryote Ratio (VPR), bacterial production (BP), viral production (VP), Viral mediated mortality (% VMM), Viral turn over time (VTT), Viral Lytic Pressure (VLyP), % of PA lysed, % of BP lysed, and Carbon released (C- Released).**
