# Supplementary material for: Viral-Induced Mortality of Prokaryotes in a Tropical Monsoonal Estuary
Source: Front Microbiol. 2017 May 23;8:895. doi: 10.3389/fmicb.2017.00895 (PMC5440509; doi:10.3389/fmicb.2017.00895)
Supplement: Supplementary file 2 [file Table2.DOCX]

|  | **Parameters** | **S1** | **S2** | **S3** | **S4** | **S5** |
| --- | --- | --- | --- | --- | --- | --- |
| **PRM)S)** | **VA** | 1.45 ± 0.77 | 1.68 ±0.67 | 1.08 ±0.36 | 1.39 ±0.65 | 1.19 ±0.72 |
|  | **BA** | 1.45 ±0.94 | 1.40 ±0.87 | 1.08 ±0.49 | 1.95 ±0.48 | 1.06 ±0.71 |
|  | **TVC** | 6.88 ± 3.09 | 4.46 ±1.99 | 7.01 ±2.25 | 3.18 ±1.29 | 4.50 ±1.45 |
|  | **VBR** | 11.80 ±6.15 | 15.61 ±10.85 | 11.70 ±5.74 | 14.56 ±4.33 | 12.37 ±4.26 |
|  | **BP** | 87.10 ±22.69 | 58.88 ±19.77 | 85.1 ±34.07 | 21.25 ±6.77 | 49.09 ±15.80 |
|  | **VP** | 4.20 ±1.60 | 15.94 ±3.66 | 5.21 ±1.64 | 16.78 ±16.21 | 4.94 ±1.25 |
|  | **VMM** | 16.79 ±6.39 | 23.44 ±13.94 | 7.45 ±4.05 | 20.83 ±6.57 | 11.76 ±5 |
|  | **VTT** | 0.37 ±0.19 | 0.89 ±1.88 | 0.23 ±0.11 | 0.73 ± 0.61 | 0.54 ±0.31 |
|  | **VLyP** | 0.98 ±0.35 | 2.35 ±1.84 | 1.47 ±0.91 | 7.3 ±4.30 | 1.27 V0.64 |
|  | **%BA LYSED** | 13.95 ±6.49 | 38.47 ±16.20 | 21.82 ±9.84 | 33.84 ±15.97 | 13.39 ±7.45 |
|  | **%BPLYSED** | 3.93 ±1.38 | 9.57 ±7.19 | 5.88 ±3.64 | 29.20 ±17.19 | 5.06 ±2.55 |
|  | **C-RELEASED** | 80.59 ±30.67 | 112.5 ±66.89 | 99.97±31.53 | 142.9 ±77.81 | 56.47 ±23.98 |
| **PRM(N)** | **VA** | 1.58 ± 0.94 | 1.80 ±1.02 | 1.35 ±0.67 | 0.88 ±0.24 | 1.15 ±0.38 |
|  | **BA** | 1.17 ±0.56 | 1.48 ±0.69 | 1.23 ±0.49 | 0.89 ±0.27 | 1.02 ±0.47 |
|  | **TVC** | 3.43 ±3.09 | 1.97 ±1.63 | 3.16 ±2.74 | 2.31 ±2.05 | 2.18 ±1.87 |
|  | **VBR** | 25.94 ±26.15 | 27.15 ±23.25 | 12.65 ±10.5 | 19.12 ±13.96 | 25.77 ±25.1 |
|  | **BP** | 77.5 ± 68.96 | 42.90 ±36.43 | 54.91 ±47.7 | 25.32 ±17.13 | 17.01 ±7.92 |
|  | **VP** | 10.38 ±9.57 | 13.63 ±4.13 | 8.46 ±8.92 | 14.70 ±5.75 | 11.36 ±6.91 |
|  | **VMM** | 11.22 ±3.75 | 25.05 ±8.11 | 11.16 ±6.03 | 17.15 ±11.11 | 14.37 ±8.80 |
|  | **VTT** | 0.35 ±0.23 | 3.58 ±3.38 | 0.23 ± 0.19 | 3.87 ±3.54 | 0.39 ±0.30 |
|  | **VLyP** | 1.21 ±1.49 | 4.21 ±4.66 | 1.44 ±1.41 | 4.32 ±3.17 | 1.60 ±0.94 |
|  | **%BA LYSED** | 6.58 ±6.12 | 16.46 ±12.31 | 3.89 ±2.42 | 16.78 ±10.72 | 7.47 ±4.19 |
|  | **%BPLYSED** | 28.83 ±24.31 | 55.78 ±51.58 | 38.21 ±35.7 | 51.41 ±42.92 | 19.79 ±17.7 |
|  | **C-RELEASED** | 42.61 ±35.68 | 72.21 ±57.54 | 22.81 ±14.9 | 77.32 ±58.01 | 22.1 ±20.36 |

**Supplementary Table:2 Table showing average ± standard deviation of various parameters at stations, S1, S2, S3, S4, and S5 during pre-monsoon season, for spring [PRM (S)] and neap [PRM (N)] tides separately. Abbreviations used are viral abundance (VA), prokaryotic abundance (PA), Viable Bacterial count (TVC), virus to prokaryote Ratio (VPR), Bacterial Production (BP), viral production (VP), Viral mediated mortality (% VMM), Viral turn over time (VTT), Viral Lytic Pressure (VLyP), % of PA lysed, % of BP lysed, and Carbon released (C- Released).**
