# Supplementary material for: Viral-Induced Mortality of Prokaryotes in a Tropical Monsoonal Estuary
Source: Front Microbiol. 2017 May 23;8:895. doi: 10.3389/fmicb.2017.00895 (PMC5440509; doi:10.3389/fmicb.2017.00895)
Supplement: Supplementary file 3 [file Image1.PDF]

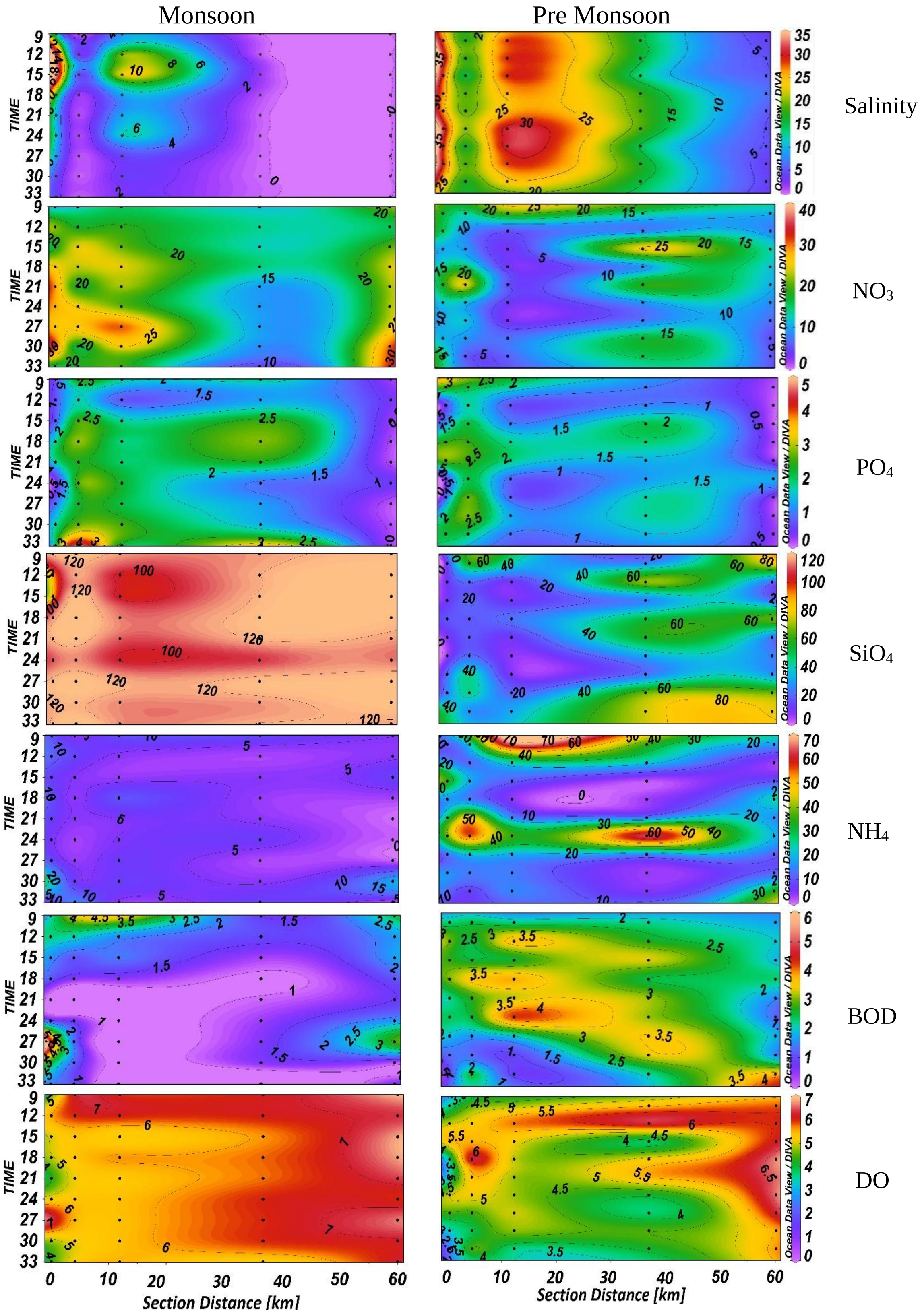

Supplementary Figure: 1 Distribution of physicochemical variables in the CE. The vertical panel represents monsoon (MON) and pre-monsoon (PRM). The horizontal panels represent various physico-chemical parameters such as salinity, Nitrate (µM), Phosphate (µM), Silicate (µM), NH_4 (µM), BOD (mg/l), and DO (mg/l). X-axis represent stations (marked as vertical dotted lines) based on distance of stations from inlet 1 (S1). Y-axis represent the time intervals for a 24 hr tidal cycle and Z-axis represent different parameters in colour contours.
